# Forehead Tactile Hallucination Is Augmented by the Perceived Risk and Accompanies Increase of Forehead Tactile Sensitivity

**DOI:** 10.3390/s21248246

**Published:** 2021-12-10

**Authors:** Jeonghee Kim, Derrick Knox, Hangue Park

**Affiliations:** 1 Department of Engineering Technology and Industrial Distribution, Texas A&M University, College Station, TX 77843, USA; jeonghee.kim@tamu.edu; 2 Department of Electrical and Computer Engineering, Texas A&M University, College Station, TX 77843, USA; derrickknox@tamu.edu; 3 Department of Multidisciplinary Engineering, Texas A&M University, College Station, TX 77843, USA

**Keywords:** neuroscience, rehabilitation, tactile hallucinations, forehead sensation, tactile hallucination, tactile sensitivity

## Abstract

Tactile hallucinations frequently occur after mental illnesses and neurodegenerative diseases like Alzheimer’s and Parkinson’s disease. Despite their common occurrence, there are several complicating factors that make it difficult to elucidate the tactile hallucinations. The forehead tactile hallucination, evoked by the physical object approaching to the forehead, can be easily and consistently evoked in healthy-bodied subjects, and therefore it would help with investigating the mechanism of tactile hallucinations. In this pilot study, we investigated the principles of the forehead tactile hallucination with eight healthy subjects. We designed the experimental setup to test the effect of sharpness and speed of objects approaching towards the forehead on the forehead tactile hallucination, in both a physical and virtual experimental setting. The forehead tactile hallucination was successfully evoked by virtual object as well as physical object, approaching the forehead. The forehead tactile hallucination was increased by the increase of sharpness and speed of the approaching object. The forehead tactile hallucination also increased the tactile sensitivity on the forehead. The forehead tactile hallucination can be solely evoked by visual feedback and augmented by the increased perceived risk. The forehead tactile hallucination also increases tactile sensitivity. These experimental results may enhance the understanding of the foundational mechanisms of tactile hallucinations.

## 1. Introduction

A tactile hallucination is an abnormal or false sensation of touch or perception of movement on the skin or inside the body [1,2]. It often accompanies an extreme level of physical and psychological distress, which are hard to overcome, even with intense physical and psychological therapy [3,4]. Examples of tactile hallucinations include the feeling of bugs crawling on the skin, internal organs moving around, or stinging of the skin [5]. Mental and neurodegenerative diseases are the main causes of tactile hallucinations. For example, ~25% people with schizophrenia suffer from tactile hallucinations [6]. Lewy body dementia, Parkinson’s disease, and Alzheimer’s disease also cause tactile hallucinations in many different forms [7,8,9,10].

Although these kinds of tactile hallucinations significantly lower people’s quality of life, available solutions to this problem are fairly limited. For example, antipsychotics drugs are not effective for 25–30% of people in treating tactile hallucinations and often come with multiple kinds of side effects [4]. They are often not pathological, and psychological coping strategies without medication can be more effective [11]. Novel treatment methods are also being investigated, such as a mirror therapy. A mirror therapy suppresses the phantom limb sensation, one type of tactile hallucination evoked after amputation. It shows amputee patients a reflection of an intact limb where their amputated limb used to be, in order to provide them with a visual scene of original body organization [12,13,14]. However, its effectiveness on a wide spectrum of patients is still inconclusive [15]. Embodiment illusion also has a potential to modulate the tactile sensitivity and suppress the tactile hallucinations, by controlling the level of body ownership and updating the body schema regarding the site causing hallucination problem [16,17,18]. In general, there is as of yet no proven effective treatment for uncomfortable tactile hallucinations, even though multiple interventions successfully modulate sensory feedback [19,20,21].

To design a therapeutic solution to overcome the tactile hallucinations, understanding its underlying principle is critical. Without clarifying the principle of generating tactile hallucinations, the promise of addressing those undesirable tactile hallucinations will remain uncertain. As the generation of the tactile hallucinations is a heavily psychological process, and there are even cases of purely psychological tactile hallucinations [22], it is important to test a psychological factor on the formation of the tactile hallucinations.

In this pilot study, we especially focused on the effect of the risk perception, as the risk notification would be one of the most important tasks for sensory signaling. Some amputee patients have reported that they have phantom sensations as if their missing limb is positioned with the posture at the event of limb damage, and the phantom pain grows stronger when the other limb is positioned with a similar posture. It has been hypothesized that this is a way for the body to avoid repeating whatever “risky” behavior that it believes will minimize the possibility of losing the other limb [23].

**Figure 1:**
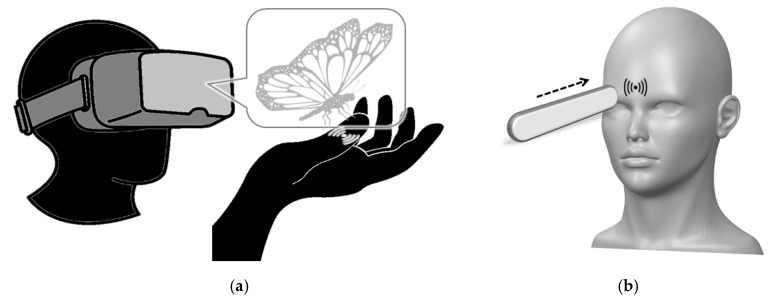
(**a**) Imaginary tactile hallucinations on the finger evoked by the object approaching to the finger in virtual (or augmented) reality and (**b**) forehead tactile hallucination evoked by the physical object approaching to the forehead. (**a**) is a hypothetical example that may not happen for the general public and (**b**) is a practical example that happens for most of the general public.

Unfortunately, the experimental investigations of the principles of tactile hallucinations have been limited, and it is hard to test the hypotheses over the tactile hallucinations. For example, it is hard to consistently and repeatedly create the same level of tactile hallucinations in each trial, even for the people who are suffering from the tactile hallucinations. Furthermore, complex neural reorganization occurs after the mental and neurodegenerative diseases that caused tactile hallucinations, which further hampers the investigation of the principle of the tactile hallucination [24]. Further, the on-going neural changes and following symptoms of mental and neurodegenerative diseases complicate the generation of tactile hallucinations. Heavily psychological tactile hallucinations, such as strong tactile feedback caused by specific visual scenes and innate fear, depend too much on psychological factors and do not work consistently in a research environment [25]. Figure 1a,b describe examples of tactile hallucinations in different settings: Figure 1a is an imaginary tactile hallucination on the finger evoked by the virtual (or augmented) object approaching, and Figure 1b is a forehead tactile hallucination evoked by the physical object approaching to the forehead.

To address the limitations in investigating the principles of the tactile hallucinations, we propose to use the forehead tactile hallucination, which is a visually-evoked tactile hallucination on the forehead by an object approaching towards the forehead. This sensation is generally described as a tingling sensation in the middle of the forehead when a pointy object, such as a pencil, is approaching the forehead [26]. It has also been used in water torture [27,28], where a person is constrained to a table and drops of water are dripped continuously on the forehead of the person. This is a kind of torture that can allegedly drive people insane, based on the strong forehead tactile hallucination. This sensation is also known to be evoked very consistently and repetitively, with minimal adaptation. Therefore, we can test multiple interventions in evoking the tactile hallucinations. We admit that this forehead tactile hallucination cannot fully explain the existing tactile hallucinations, as the principles of tactile hallucinations varies. However, it would be important to gain insight into the tactile hallucinations, using the experiment-friendly tactile hallucinations.

To investigate the effect of perceived risk on the forehead tactile hallucination, we designed three tests with three hypotheses, one for each test. *The first is to test the hypothesis* that visual feedback of an object approaching the forehead is enough to evoke the tactile hallucinations on the forehead without any physical interaction. As physical objects close to the forehead may have a tiny physical effect from airflow or touching the hair on the forehead, we employed virtual reality (VR) that is completely free from physical interaction. *The second is to test the hypothesis* that the forehead tactile hallucination will be augmented by the perceived risk. We expect that the level of perceived risk would play an important role in psychological process of the forehead tactile hallucination. We modulated the perceived risk by the sharpness and speed of the object approaching the forehead. *The third is to test the hypothesis* that the tactile hallucinations evoked on the forehead will change the tactile sensitivity on the forehead. The actual sensory feedback and the tactile hallucinations will interact with each other at the central nervous system (CNS), and therefore we expect the reciprocal effect between each other.

## 2. Materials and Methods

We designed the experimental setup to test the three hypotheses using two different types of physical and virtual objects (a standard mechanical pencil and a stick eraser) approaching towards the forehead in different speeds.

### 2.1. Human Participants

The experiments in this pilot study were performed in accordance with relevant guidelines and regulations, according to the procedure described in the protocol approved by the Institutional Review Board of Texas A&M University (IRB2018-1497D; date of approval as 11 April 2019). Informed consent was collected from all subjects. Eight healthy human subjects with no history of neurological disorders participated in the experiments in this pilot study. The subject group consisted of one female and seven males. The age of subjects ranged from 20 to 40 with mean age of 27.

### 2.2. Experimental Setup

#### 2.2.1. Physical Objects

The pencil used for the test was a standard mechanical pencil with a thin lead. The length of the body was 14.3 cm and the diameter of the sharp lead of the pencil was 0.7 mm. During the experiment, the sharp lead was extended to 30 mm from the tip of the pencil to create an extremely pointy outlook. The stick eraser used for the test was a standard pen-type stick eraser with a refillable eraser lead. The length of the body was 12.7 mm and the diameter of the eraser lead was 9 mm. During the experiment, the eraser lead (rubber portion) was extended to 30 mm from the tip of the stick eraser.

#### 2.2.2. Virtual Objects

Oculus Rift (Facebook Technologies, LLC, Menlo Park, CA, USA) was selected as the VR headset during the experiment. A VR interface was created based on a Unity real-time VR development platform (https://unity.com/ (accessed on 8 December 2021)), to present either a pencil or a stick eraser approaching the subject’s forehead. Both pencil and eraser were designed to sit at the level of the subject’s eye and point to the top of their nose (i.e., forehead). Both pencil and eraser were modeled to look similar to the selected pencil and eraser in the experiment.

#### 2.2.3. Method of Object Approach

Both physical and virtual objects moved at two speeds, by the operator and the program, respectively. We set 15 cm/s as a normal speed and 45 cm/s as a fast speed, as the former takes ~3 s to approach from ~1.5 feet distance while the latter take ~1 s. Operators practiced a number of times with a metronome to move the object consistently at both speeds of 15 cm/s and 45 cm/s. However, note that, no metronome sound was used during the actual experiment and there is a potential error in both approaching speeds.

### 2.3. Evaluation Metrics

#### 2.3.1. Scale of the Tactile Hallucination

**Figure 2:**
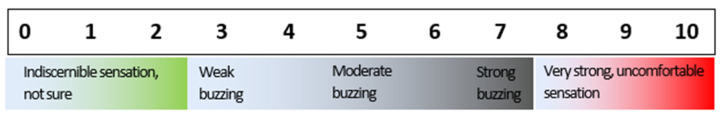
Ten-point Likert scale used in the pilot study to quantify the subjects’ forehead tactile hallucination.

A ten-point Likert scale was used to quantify the tactile hallucination at forehead. Subjects were asked to take a seat in an office chair in the laboratory. The operator then turned the chair for subjects to face against a white wall in the lab. Subjects were asked to maintain a good upright posture during the entire experiment. Before the experiments began, subjects were also introduced to the ten-point Likert scale that was being used to evaluate their perception. The grading standard was a level from zero to ten, as zero being feeling nothing at all and five as clear sensation, while the strength of sensation increases as the number becomes larger (see Figure 2). If the sensation was too strong and disturbing, subjects were instructed to select a level from six to ten, with six being minimally disturbing and ten being extremely disturbing. At the end of each trial for Experiment I and II (see Section 2.4.1 and Section 2.4.2, respectively), subjects were asked to answer their perceived sensation using this Likert scale from 0 to 10.

#### 2.3.2. Scale of the Tactile Sensitivity

We used a standard Von Frey hair test [29] to quantify the tactile sensitivity. The force was increased with minimal resolution of the Von Frey hair test kit (20-piece Touch Test^®^ Sensory Evaluators, North Coast Medical) until subjects feel the hair touching their forehead. The procedure was repeated until the operator found the minimum force evoking the tactile feedback on the forehead. At the end of each trial for Experiment III (see Section 2.4.3), subjects were asked to answer their tactile sensitivity using this Von Frey hair test kit.

### 2.4. Experiment Design

#### 2.4.1. Experiment I

**Figure 3:**
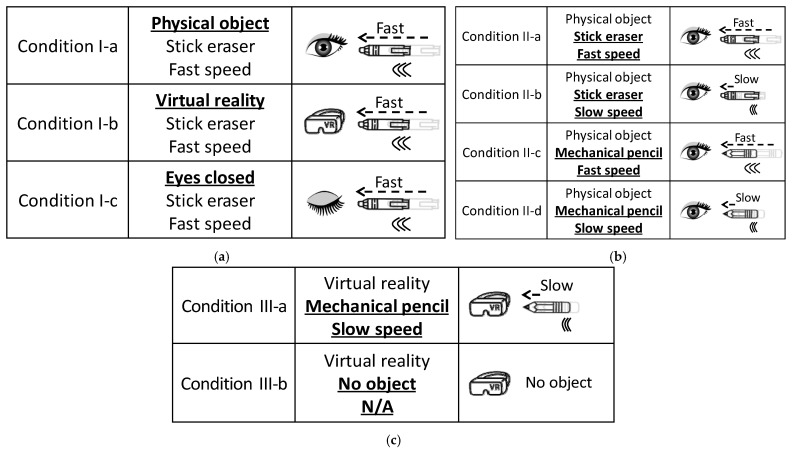
Experimental conditions for the (**a**) Experiment I testing the effect of visual feedback and virtual reality, (**b**) Experiment II testing the effect of perceived risk, and (**c**) Experiment III testing the tactile sensitivity change.

The first experiment was designed to test the first hypothesis that “visual feedback of the object approaching the forehead will evoke the tactile hallucination on the forehead without any physical interaction”. Its test conditions are summarized in detail in Figure 3a. For the data integrity, each test condition was repeated twice in a random order, with 60-s intervals in between.

To test the first hypothesis, we compared subjects’ perception on the forehead for three different conditions: (1) eyes open with a physical pen-type eraser (stick eraser) approaching the forehead, (2) eyes open with a virtual stick eraser approaching the forehead (at VR), and (3) eyes closed with a physical stick eraser approaching the forehead. The approaching speed was selected as 45 cm/s (i.e., fast speed), for the forehead tactile hallucination to still be evaluated clearly, in case it was attenuated at the virtual reality environment. The operator or virtual reality program moved the object from a distance of ~45 cm from the forehead to a distance of ~1 cm from the forehead, steadily over 1 s (~45 cm/s). Note that the operator or virtual reality program then stopped clearly before physical contact was made, and took the object away from the forehead.

**Figure 4:**
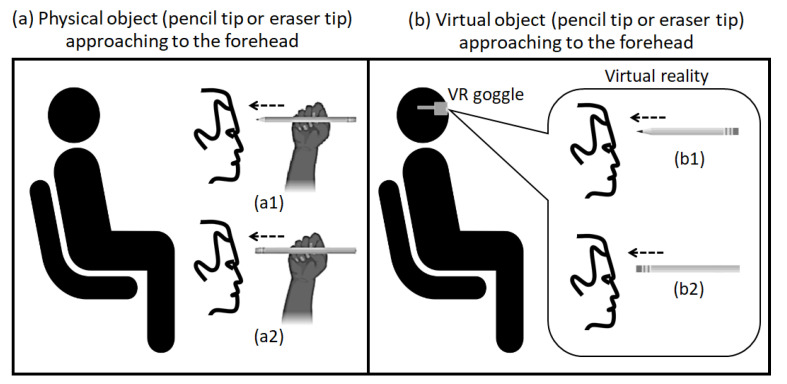
A mechanical sharp pencil with its pencil tip or a stick eraser with its eraser tip moving towards the subject’s forehead. (**a**) describes the test condition with physical object approaching to the subject’s forehead (**a1** with pencil and **a2** with eraser) and (**b**) describes the test condition with virtual object (with VR goggle) to the subject’s forehead (**b1** with pencil and **b2** with eraser).

In an open-eye condition, subjects were asked to focus on the object moving towards their forehead, as shown in Figure 4a,b. In a closed-eye condition, subjects were asked to close their eyes when they were asked by the operator, and the operator gave a verbal 3-s countdown before the tests (“3, 2, 1, go”), and subjects were asked to open their eyes after the object was taken away from the forehead. All subjects were asked to report the forehead sensation on the ten-point Likert scale (Figure 2), at each test condition right after the object was taken away from the forehead.

#### 2.4.2. Experiment II

To test the second hypothesis that “the forehead tactile hallucination will be augmented by the perceived risk”, the second experiment was performed with real physical objects and repeated with changing object sharpness and approaching speed (eyes open for all conditions). Its test conditions are described in detail in Figure 3b. First, the sharpness of the object changes by switching the object between the mechanical pencil and the stick eraser. Second, the approaching speed was selected between normal (15 cm/s) and fast (45 cm/s) speeds. We expected both the sharper physical object and the faster speed will augment the perceived risk.

For data integrity, all test conditions were applied in a random order using a random number generator and twice per condition. Between each test, subjects were told to wait and relax for 60 s, to minimize the habituation or adaptation of the nervous system to the forehead stimulus. All subjects were asked to report the forehead sensation on the ten-point Likert scale (Figure 2), at each test condition right after the object was taken away from the forehead.

#### 2.4.3. Experiment III

The third experiment was designed to test the third hypothesis that “the tactile hallucination evoked on the forehead will change the tactile sensitivity on the forehead.” Its test conditions are described in detail in Figure 3c. Subjects were asked to wear the VR headset, and wear a hairband to pull their hair back and expose their forehead at the top of the VR goggle. At two different conditions, with or without virtual object approaching to the forehead, the operator conducted the Von Frey hair test to determine their tactile sensitivity on the forehead. Note that the mechanical pencil and slow speed (i.e., 15 cm/s) were selected, because we wanted the tactile hallucination strong enough while having enough time to evaluate the forehead tactile sensitivity. Furthermore, note that the operator gave a verbal 3-s countdown before the tests (“3, 2, 1, go”), with or without virtual object approaching to the forehead, to provide an auditory cue of the object approaching the forehead.

The sensitivity was evaluated by the minimal force that subjects can detect on their forehead in each condition. Since the subjects wore the VR headset with the hairband, we applied the tactile sensitivity test right above the target location where the virtual object was approaching. The Von Frey hair test was applied to subject’s forehead at random timing and was not applied sometimes, so that subjects could not report the hair touching their forehead by expectation. For the data integrity, each test condition was repeated twice in a random order, with 60 s intervals in between.

### 2.5. Statistical Analysis

To determine the efficacy of the independent factors on dependent variable, and to account for both within-subject and across-subject variability, we performed a linear mixed model analysis (SPSS, IBM, Chicago, IL, USA) per each experimental result. Both subjects and trials were set as random factors, commonly for all three analyses for each of the three experiments. For the independent factors, their effects on dependent variables were determined both independently and in combination. For example, the availability of visual feedback (eyes open/close) and the type of object (physical/virtual) were set as independent factors, with the intensity of forehead sensation as a dependent variable. For Exp. II, the approaching speed and the sharpness of the object were set as independent factors, with the intensity of forehead sensation as a dependent variable. For Exp. III, the existence of approaching object was set as an independent factor, with the sensitivity threshold as a dependent variable. The significance level was set at 0.05. All statistical data were represented as Mean ± SE (standard error) in the results section and all statistical comparisons were represented with the corresponding *p* values.

## 3. Results

### 3.1. Experiment I

**Figure 5:**
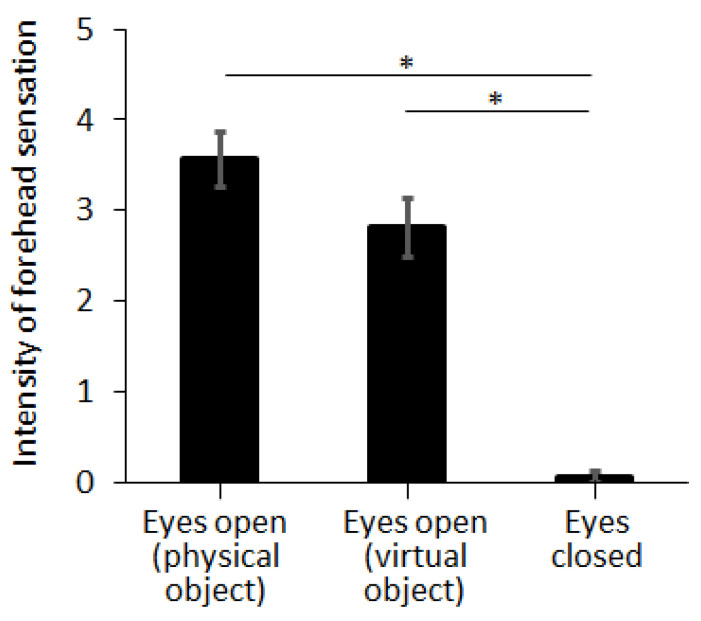
Tactile hallucination evoked by physical or virtual stick eraser approaching to the forehead at fast speed (45 cm/s). Three conditions of visual feedback were tested: open-eyes with physical object, virtual-reality condition with virtual object, and closed-eyes with physical object (see Figure 3a). Error bar indicates the standard error and asterisk (*) indicates statistical difference with 95% confidence interval.

In Exp. I, we compared the intensity of the forehead tactile hallucination according to the condition of visual feedback, by using virtual reality and closing the eyes. Intensity of forehead sensation was evaluated as 3.56 ± 0.30, 2.81 ± 0.32, and 0.06 ± 0.06 for eyes open with a physical object, eyes open with a virtual object, and eyes closed with a physical object, respectively (Figure 5). Note that the stick eraser was selected as the physical or virtual object and approaching speed was selected as 45 cm/s (i.e., fast approaching speed). The intensity of the forehead sensation was not different between the cases of physical object and virtual object (*p* = 0.098, η^2^ = 0.088). The intensity of forehead sensation was significantly decreased by closing the eyes (*p* < 0.001, η^2^ = 0.811).

Note that we screened subjects by asking if they had any experience of motion sickness in both physical and virtual environments. We excluded subjects who answered “yes” to this question. No subject reported dizziness or cybersickness after the completion of the experiment. However, we cannot exclude the possibility that symptoms of cybersickness may have not been identified, as we did not implement a cybersickness questionnaire [30].

### 3.2. Experiment II

**Figure 6:**
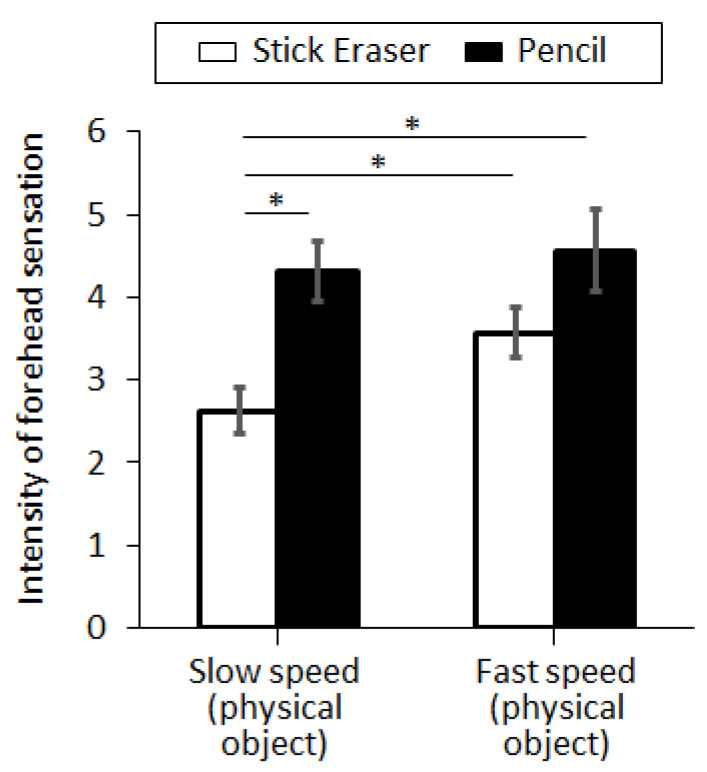
Tactile hallucination evoked by the physical stick eraser or the physical pencil approaching to the forehead. Four conditions of perceived risk were tested: normal approaching speed with physical stick eraser, normal approaching speed with physical pencil, fast approaching speed with physical stick eraser, and fast approaching speed with physical pencil (see Figure 3b). Error bar indicates the standard error and asterisk (*) indicates statistical difference with 95% confidence interval.

In Exp. II, we compared the intensity of forehead sensation according to the perceived risk, which was controlled by the sharpness and approaching speed of the object. The results of the perceived sensation were displayed in Figure 6. With the normal approaching speed (15 cm/s), the intensity of the forehead sensation was evaluated as 2.63 ± 0.27 and 4.31 ± 0.36 for the stick eraser and the mechanical pencil, respectively. With the fast-approaching speed (45 cm/s), the intensity of the forehead sensation was measured as 3.56 ± 0.30 and 4.56 ± 0.49 for the stick eraser and the mechanical pencil, respectively (see Figure 6). The intensity of forehead sensation was larger with the mechanical pencil than the stick eraser, at normal approaching speed (*p* = 0.001, η^2^ = 0.317) but not at fast approaching speed (*p* = 0.093, η^2^ = 0.091). The intensity of forehead sensation was also larger with the fast-approaching speed than with the normal approaching speed, with the stick eraser (*p* = 0.028, η^2^ = 0.151). However, the intensity was not different between the two speed settings when the mechanical pencil was used (*p* = 0.685, η^2^ = 0.006).

### 3.3. Experiment III

**Figure 7:**
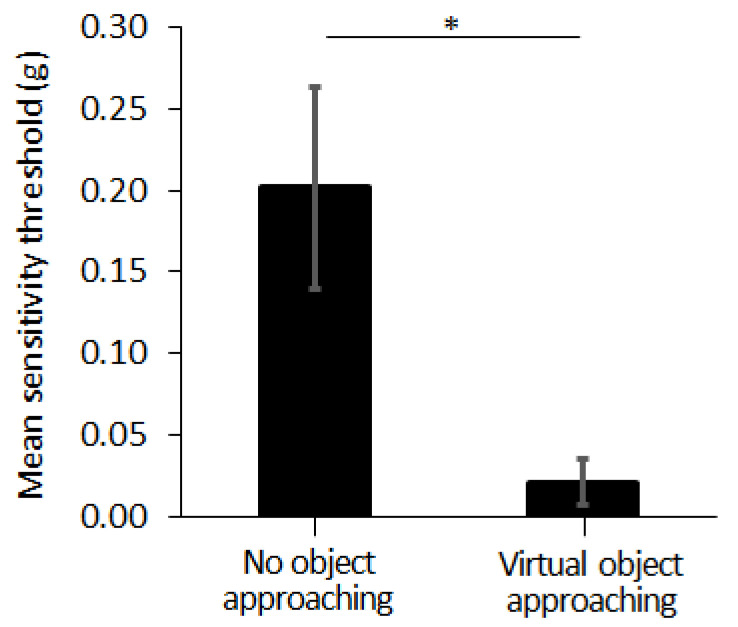
Sensitivity threshold on the forehead with and without the virtual stick eraser approaching to the forehead (see Figure 3c). Error bar indicates the standard error and asterisk (*) indicates statistical difference with 95% confidence interval.

In Exp. III, we compared the tactile sensitivity on the forehead, according to the existence of the approaching object, at VR environment. The tactile sensitivity, evaluated by the tactile threshold, was measured as 0.02 ± 0.01 (g) and 0.20 ± 0.06 (g), with and without the virtual object approaching towards the forehead, respectively (see Figure 7). The threshold value of the tactile sensitivity was smaller when the virtual object was approaching the forehead, compared to the case when no virtual object is approaching the forehead (*p* = 0.046, η^2^ = 0.492).

## 4. Discussion

### 4.1. Forehead Tactile Hallucination Is Evoked by Visual Feedback

Visual feedback solely evoked the sensation on subjects’ foreheads without any need of physical interaction, which supports our first hypothesis that visual feedback is enough to evoke the tactile hallucination on the forehead without any physical interaction. A virtual object approaching the forehead successfully evoked the same level of intensity of tactile hallucination on the forehead as a physical object approaching the forehead. These results of Exp I support the idea that forehead sensation by the approaching object is a tactile hallucination regardless of the physical interaction.

Moreover, when subjects closed their eyes, the intensity of the tactile hallucination was clearly decreased and close to zero. Considering that the operator still provided a verbal signal (“3, 2, 1, go”) before positioning the pointed object onto the forehead, this result suggests that imagination through the auditory feedback is not enough to evoke the forehead tactile hallucination; thus, visual feedback is necessary. Based on the fact that visual feedback has a strong effect on forehead tactile hallucination, we expect that efference copy and following sensory reafference play an important role in evoking tactile hallucinations. The conventional comparator model explains the perception by two primary factors—actual stimulus and sensory reafference [31]. If the tactile hallucination was perceived with no actual stimulus, it may have been created by a change in sensory reafference. Previous works have found that it is possible for visual feedback through virtual reality to alter tactile sensations, which suggests that there is a link between visual feedback and sensory reafference [32].

### 4.2. Forehead Tactile Hallucination Becomes Stronger as the Level of Perceived Risk Increases

As the object approaching towards the forehead became sharper or the object approaches with faster speed, subjects felt stronger forehead tactile hallucination. As the sharper objects will be perceived with higher risk than dull objects, and the faster-approaching objects will be perceived with higher risk than slow-approaching objects, this result suggests that the perceived risk is an important factor in augmenting the forehead tactile hallucination. When one of the two risk factors exist (i.e., fast approaching speed or sharp object), the other risk factor did not change the forehead tactile hallucination, which suggests that either speed or sharpness was enough to increase the perception of risk.

These results support our second hypothesis that the forehead tactile hallucination will be augmented by the perceived risk. We interpret this result as the nervous system providing a warning signal when faced with a threat. As tactile feedback is a more intuitive sensation than visual feedback, with less processing needs and processing delay [33], the nervous system may activate tactile feedback for better preparation of the body to the potential threat. The prior studies on phantom limb pain for amputee patients also suggest that the nervous system provides a warning signal to not repeat the mistake to lose the limb [23]. This result also suggests that the nervous system does not always suppress the unpleasant sensations and rather uses them if needed. As unpleasant sensations are usually suppressed by adaptation of sensory receptors and changed sensory pathway gain at the thalamus or somatosensory cortex, this process of suppression may be inactivated by the generation of tactile hallucination.

### 4.3. Tactile Sensitivity of the Forehead Increases with the Forehead Tactile Hallucination

When the virtual object was approaching subjects’ foreheads, subjects reported higher tactile sensitivity on their forehead compared to the case of no approaching object. These results support our third hypothesis that the tactile hallucination evoked on the forehead will change tactile sensitivity on the forehead. This suggests that actual sensory feedback and the tactile hallucination closely interact with each other. If the two are closely intertwined, then it may be possible to modulate the tactile hallucination with the physical sensory interaction too. Furthermore, it would be helpful to advance the sensory feedback model by integrating tactile hallucinations. Building a theoretical model that explains tactile hallucinations with the normal sensation by physical interaction will help us come to a deeper understanding of tactile hallucinations, similar to the case of the VR modulating subjects’ real-world tactile sensitivity.

### 4.4. Our Result Is Linked with the Existence of the Occipital-Cerebellar-Cortical Loop

We also speculate that the occipital-cerebellar-cortical loop may be involved in generating the tactile hallucination. Visual feedback from the occipital lobe would spur the creation of an efferent signal in the cerebellum, which would be delivered directly to the somatosensory cortex. This occipital-cerebellar-cortical loop would generate feelings of touch independently of the thalamus and the peripheral nervous system, which may explain the reason for tactile hallucinations. There is also growing evidence explaining how sensory gain can be modulated by the somatosensory cortex, as well as the thalamus [34], which can be linked with our experimental result that the strength of the tactile hallucination was modulated by perceived risk without any physical interaction and evoked sensory feedback.

### 4.5. Great Potential of the Forehead Tactile Hallucination to Be Used in the Study of the Tactile Hallucination

Tactile hallucination is a multivariate function affected by psychological conditions, as well as visual feedback, and therefore it is hard to design the experiment and get the reliable result. For example, phantom limb sensation is unique to the amputees and happens unexpectedly with random timing. In one study of phantom limb pain (n = 183), 81% of the patients suffering from phantom sensations described their pain as “episodic”, and 50% reported one episode of phantom pain per week or less [35]. Accordingly, the study of the phantom sensation has been extremely limited [36]. The forehead tactile hallucination is a promising candidate to be used for the study of tactile hallucinations, as it can be easily evoked by the pointy object approaching the forehead and observed in most people [37]. Through our experiment, we could also confirm that the forehead tactile hallucination could be consistently evoked for subjects. The ability to evoke the sensation at any time among most healthy and able-bodied subjects, in a consistent manner, simply by the approach of a pointy object to the forehead, makes this approach ideal for future use in investigation of the tactile hallucination.

### 4.6. Limitation of the Study

As the limited number of subjects (8) poses a question in the reliability of the conclusions made in this paper, this study needs to be understood as a pilot study. To overcome the potential error caused by the psychological effect as well as the biological variation, we will recruit larger number of subjects in the next study. Further, there is potential aftereffect that has not been fully considered in the study design. Although we provided 60-s intervals in between the test conditions and the experimental order was fully randomized, the order of each condition was not counterbalanced among subjects. In the future experiment, it would be important to counterbalance the order of each condition among subjects and check the aftereffect with independent subject group.

## 5. Conclusions

In this pilot study, we investigated the principles of the forehead tactile hallucination, regarding the effect of the perceived risk and the resulting changes in tactile sensitivity. Perceived risk increased the strength of the forehead tactile hallucination, and the forehead tactile sensitivity was increased with the forehead tactile hallucination. We expect that the forehead tactile hallucination would provide a new experimental framework for tactile hallucination study and that the presented experimental results would provide insights for the researchers to further investigate the solutions to suppress the tactile hallucinations.

## Data Availability

The datasets generated and/or analyzed during the current study are available from the corresponding author on reasonable request.
